# Surgical, orthodontic, and prosthetic management of amelogenesis imperfecta associated with severe open bite: a case report

**DOI:** 10.25122/jml-2024-0259

**Published:** 2024-10

**Authors:** Amel Labidi, Sana Bekri, Yosra Mabrouk, Sameh Rzigui, Ines Dallel, Ramzi Moatemri, Lamia Mansour

**Affiliations:** 1 Department of Removable Prosthodontics, Faculty of Dental Medicine, University of Monastir, Monastir, Tunisia; 2 Laboratory of Dento-Facial, Clinical and Biological Approach (ABCDF) (LR12ES10), Faculty of Dental Medicine, University of Monastir, Monastir, Tunisia; 3 Department of Orthodontics, University of Monastir, Monastir, Tunisia; 4 Department of Maxillofacial, Plastic, and Esthetic Surgery, Sahloul University Hospital, Sousse, Tunisia

**Keywords:** amelogenesis imperfecta, open bite, orthognathic surgery, orthodontics, dentistry, prosthodontics treatment

## Abstract

Amelogenesis imperfecta refers to a group of hereditary diseases that affect dental enamel, often leading to a wide range of clinical manifestations and aesthetic concerns. This case report describes a female patient diagnosed with amelogenesis imperfecta associated with a skeletal open bite. The treatment approach was multidisciplinary. Periodontal therapy was initiated, followed by orthodontic treatment using brackets on temporary crowns to expand and coordinate the maxillary and mandibular arches. Subsequently, orthognathic surgery, including a LeFort I osteotomy and genioplasty, was performed. The fixed prosthodontic rehabilitation performed with metal-ceramic crowns was the last step of the treatment. The final result was both functionally and esthetically satisfactory for the patient.

## INTRODUCTION

Amelogenesis imperfecta (AI) belongs to a group of hereditary disorders. Its frequency ranges from 1 in 700 and 1 in 14,000 [1]. It is characterized by defective formation or calcification of the enamel, leading to various clinical forms and appearances [2]. There are three types of AI: hypoplasia hypomaturation, hypocalcification, and hypomaturation/hypoplasia/taurodontism [3]. Several reports have described cases of AI associated with craniofacial problems, such as skeletal Class II or Class III malocclusions and open bite malocclusions [4]. Rowley *et al*. [5] reported that 20% of AI cases have severe anterior open bite. These cases are marked by disabilities in oral function, discomfort in eating, and mainly poor aesthetics, affecting the patient’s psychology and quality of life. The oro-facial and dental treatment of these cases requires close collaboration among several specialties, mainly maxillofacial, orthodontic, and prosthetic management [6]. The aim of this paper was to present a multidisciplinary approach to managing a clinical case diagnosed with amelogenesis imperfecta, which was associated with a severe open bite.

## CLINICAL REPORT

A 14-year-old female patient in good general health consulted the dental clinic of Monastir in 2014. Her main complaints were an anesthetic smile, dental sensitivity, and chewing difficulties.

### Clinical examination

**Figure 1:**
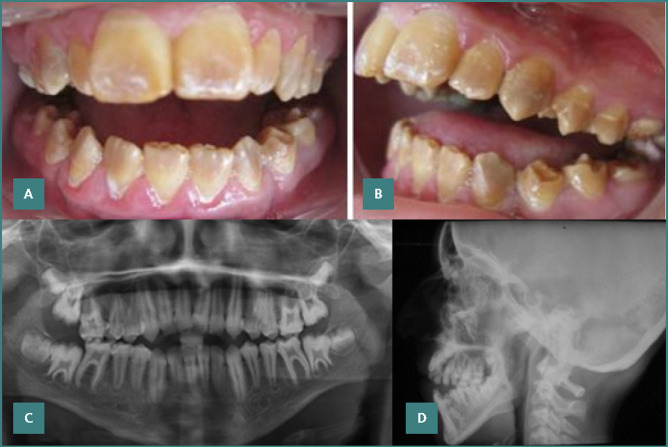
Initial Clinical presentation and radiographic assessment. A, Intraoral front view showing the yellow color of the teeth and rough enamel surfaces. B, Intraoral right lateral view revealing severe anterior open bite. C, Orhopantomographic radiograph. D, Lateral cephalogram radiograph.

On a frontal view examination, the extra-oral assessment revealed a narrow, elongated face with dark circles under the eyes, flattened cheekbones, and labial non-occlusion associated with an open bite. An increase in the height of the lower face was observed. The reference lines — the eyebrow, pupillary, and bicommissural lines — were parallel and aligned perpendicularly to the mid-sagittal plane. These features, along with other observed characteristics, suggested mouth breathing and were consistent with ‘adenoid facies’. The lateral profile examination revealed a convex facial profile characterized by a domed forehead, a straight nasal bridge, and persistent labial incompetence. The nasolabial angle was reduced (< 90°), and the gonial angle was open. A slight inferior retrocheilia was noted, along with retrogenia of the chin and a flattened labiomental sulcus. The patient’s profile was transfrontal. Intra-oral clinical examination showed the presence of permanent dentition as well as a remarkable presence of plaque due to poor oral hygiene accentuated by dental sensitivities. The teeth exhibited a yellow-brown, crumbly enamel that fractured easily from the underlying dentin, consistent with a diagnosis of hypoplastic amelogenesis imperfecta. Despite enamel defects, tooth morphology was generally intact, with no diastemas (Figure 1A). The patient presented a thick gingival phenotype and showed signs of moderate to severe gingivitis in these sites. They were favored by plaque retention factors, such as calculus and a porous tooth surface. The occlusal analysis revealed a total anterior open bite, with only the last molars in occlusion, exhibiting slight abrasion on their occlusal surfaces (Figure 1B). There was an Angle Class II molar and canine relationship bilaterally in the anteroposterior direction. Additionally, there was a misalignment of the inter-incisal points, with a 3 mm shift of the mandibular inter-incisal point to the right. The patient also had a non-functional anterior guidance due to the complete anterior open bite.

The panoramic X-ray revealed the absence of carious lesions and dental inclusion and an enamel layer that was either absent or difficult to distinguish from the dentin due to similar radiopacity. There was no evidence of bone involvement or apical radiolucency. Additionally, wide pulp chambers were observed, particularly in the first molars, attributed to the patient's young age rather than taurodontism. The X-ray also showed the presence of four impacted third molars (Figure 1C). Lateral teleradiography revealed an open gonial angle, a tapered symphysis, and a thin, backward-tilted condyle, all of which are radiological signs of posterior rotation of the mandibular condyle (facial hyper-divergence) (Figure 1D, Table 1). Based on these clinical and radiographic findings, along with a familial history of similar dental anomalies, a diagnosis of inherited hypoplastic amelogenesis imperfecta associated with a skeletal anterior open bite was made.

**Table 1:** Tweed's cephalometric analysis

	Normal Value	Clinical value before treatment	Interpretation before	Clinical value after treatment	Interpretation after
SNA	82° ± 2°	82°	Maxillary normognathia	83°	Maxillary normognathia
SNB	80° ± 2°	75°	Mandibular retrognathia	79°	Mandibular normognathia
ANB	[0 - 4°]	7°	Skeletal Class II	4°	Skeletal Class I
IMPA	87°	95°	Mandibular proalveolia	92°	Mandibular proalveolia
I/F	107° ± 5°	118°	Maxillary proalveolia	107°	Maxillary normoalveolia
FMA	25° ± 3°	46°	Facial hyperdivergent	27°	Facial normodivergent
I/i	135°	121°	Bi-proalveolia	130°	Proalveolia

SNA, Sella-Nasion-A Point Angle; SNB, Sella-Nasion-B Point Angle; ANB, Difference between SNA and SNB Angles; IMPA, Incisor Mandibular Plane Angle; FMA, Frankfort Mandibular Plane Angle

The therapeutic goals for this case were to reduce dental hypersensitivity, prevent tooth decay, maintain periodontal health, correct the anterior open bite, achieve esthetic and functional rehabilitation, and establish harmonious inter- and intra-arch relationships. Different specialties were involved in managing this clinical case, including periodontics, orthodontics, maxillofacial surgery, and prosthodontics. After obtaining the patient’s written informed consent and the ethical approval regarding the considered treatment, from the Ethics and Research Committee of the Faculty of Medicine of Monastir, Tunisia (Ethical approval number IORG 008138N°130/OMB 0990-0169, 30 January 2020), as well as consent for case publication, the treatment plan was initiated.

### Periodontal treatment

Restoring periodontal health was crucial before initiating orthodontic treatment, as moving teeth in the presence of an inflamed periodontium could exacerbate the condition or result in bone loss. In this case, the patient presented with an inflamed periodontium, as it was difficult and painful to maintain adequate oral hygiene due to hypersensitivity. Therefore, she received motivational sessions on proper brushing techniques and underwent scaling under local anesthesia to remove supra- and subgingival plaque and calculus. After a 3-month revaluation, the gingiva was healthy, and it was possible to perform the other treatment steps.

### Orthodontic treatment and orthognathic surgery

**Figure 2:**
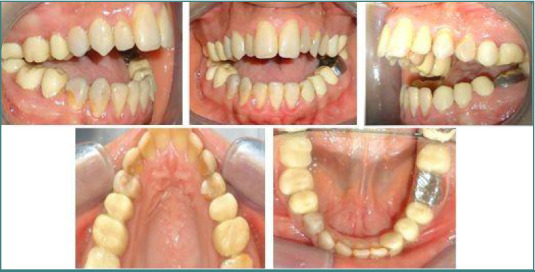
Endo-oral images of the temporary prostheses before the orthodontic phase

Given the demineralized state of the dental tissues, which was unfavorable for the placement and bonding of orthodontic brackets, the decision was made to begin with a pre-orthodontic prosthetic stage. This involved using separate temporary crowns made of auto-cured acrylic material (Tab 2000 Kerr, Kloten, Switzerland). First, minimal teeth preparation was performed using dental diamond burs. Then, acrylic resin temporary crowns were made from a wax-up based on study models, to be sealed later with temporary cement (Temp-Bond Kerr, Kloten, Switzerland) (Figure 2). The patient presented with a skeletal Class II profile (ANB angle = 7°) due to mandibular retrognathia, long face syndrome, and a severe open bite (46° confirmed by Frankfort Mandibular Plane Angle [FMA]). Additionally, she presented with upper and lower incisor proclination, Class II malocclusion with a 6 mm overjet, and an irregular open bite reaching 11 mm in the incisor region.

**Figure 3:**
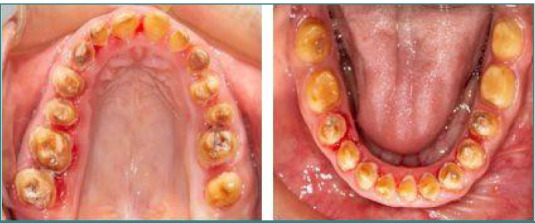
Maxillary and mandibular arch expansion following orthodontic treatment

The aim of the orthodontic treatment was to expand the maxillary and mandibular arches in order to correct the narrow V-shaped arch caused by mouth-breathing. The expansion reached 3 mm in the maxilla and 2 mm in the mandible. The coordination of the upper and lower jaws was necessary for a good occlusion after the surgical intervention in all sagittal, vertical, and transverse directions (Figure 3). The orthodontic treatment was followed by bi-maxillary orthognathic surgery. The maxilla was lowered to the anterior level using the LeFort I impaction surgical technique [7]. Mandibular surgery involved chin advancement and height reduction to address retrognathia and correct vertical dimensions [8]. The ortho-surgical treatment lasted 3 years.

### Prosthetic treatment

**Figure 4:**
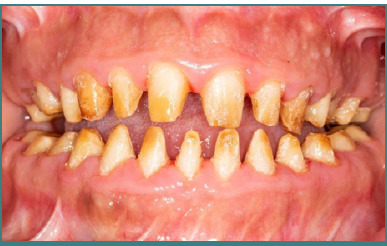
Intraoral image of the final dental preparation

**Figure 5:**
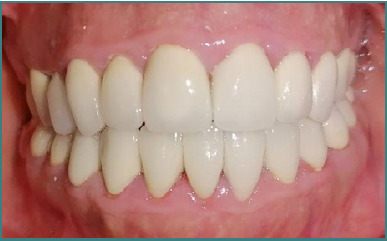
Final sealing of the prostheses

**Figure 6:**
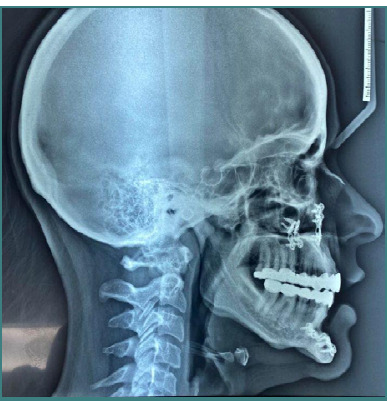
Lateral cephalometric radiograph showing the skeletal and dental structures post-treatment

**Figure 7:**
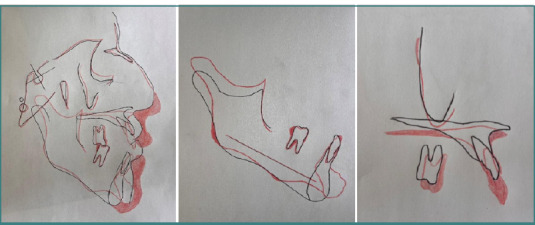
Cephalometric tracings: pre- and post-treatment comparisons

**Figure 8:**
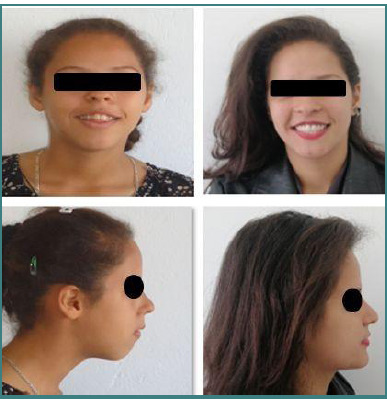
Improvement in facial esthetics, skin profile, correction of bilabial incompetence

**Figure 9:**
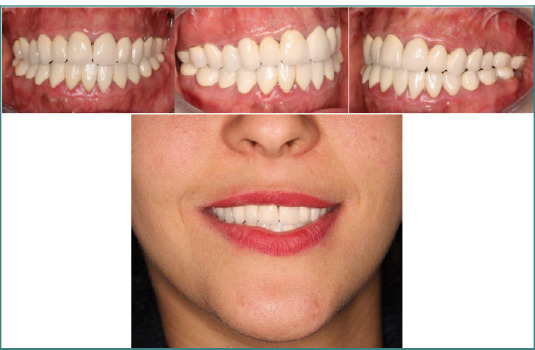
The esthetic and functional results were stable and satisfactory at the three-year post-treatment follow-up

Once the orthodontic and surgical preparations were completed and bone healing was assured, prosthetic treatment commenced. The dental preparations were refined, as they had been kept minimal during the construction of the temporary prostheses (Figure 4). The maxillary and mandibular final impressions were taken using the wash technique with high and low silicone viscosity (Protesil, Vannini Dental Industry). The global impression was cast, and bite registration was performed using a semi-adjustable articulator (Bioart), with the maxillary position transferred using a facebow. The mandibular cast was assembled in centric relation, validated through temporary prostheses. A metal framework with paired ceramic crowns was fabricated and tried in the mouth. After selecting the ceramic color, the crowns were mounted in the laboratory and tried at the biscuit stage. Following the glazing process, the final prostheses were cemented in place (Figure 5). The treatment lasted 4 years. An improvement in the endo-buccal situation and the inter- and intra-arch relationships was noted (Figures 5-7 and Table 1). The collaborative efforts successfully enhanced the patient’s esthetics and functionality (Figure 8, Table 2). Regular follow-ups were scheduled for up to 3 years post-treatment. The esthetic and functional results were stable and satisfactory for the patient and the medical team (Figure 9).

**Table 2:** Checklist of procedures performed according to overlapping specialties

**Periodontics**	Motivational session on oral hygiene guidance. Scaling session to eliminate tde supra- and sub-gingival calculus and plaque.
**Prosthodontics**	Minimal teeth preparation Sealing of resin temporary crowns to facilitate orthodontic bracket placement
**Orthodontics**	Expansion of the maxillary arch by 3 mm. Expansion of the mandibular arch by 2 mm.
**Maxillofacial surgery**	Bi-maxillary orthognathic surgery: lowering the maxilla to the anterior level using the LeFort I impaction surgical technique. Mandibular advancement using the Obwegeser-Dalpont approach and combined genioplasty
**Prosthodontics**	Fabrication and placement of metal-ceramic crowns.

## DISCUSSION

The diagnosis of amelogenesis imperfecta is based on the clinical aspect of the enamel. The radiographic features also guide the classification of AI. Several dental defects are associated with AI, such as tooth sensitivity and dental caries. Despite the enamel anomalies in AI, dental caries are not predominant, especially in the hypoplastic type [9]. As shown in the present case, the teeth affected with the hypoplastic type of AI did not present dental caries. This could be explained by the significantly high adhesion of *Lactobacillus casei* and the weak adhesion of *Streptococcus mutans* on the dental hard tissues in AI [10]. In addition to dental defects, AI is associated with skeletal anomalies, such as Class II and Class III [4]. Open bite is also frequent in AI patients. In their systematic review, Poulsen *et al*. [11] reported that open bite is the most commonly reported malocclusion in patients with AI. According to Alenka Pavlič *et al*., hypoplastic AI with a rough surface of the teeth is associated with increased vertical dimensions [12]. An early clinical diagnosis of increased vertical dimensions in patients with rough hypoplastic AI is paramount to determine the correct timing of the orthodontic intervention [12]. In this case, the open bite was so severe that only the second molars were in occlusion.

The management of such cases requires a multidisciplinary approach involving orthodontic treatment, orthognathic surgery, and prosthodontic treatment. Additionally, particular care in prevention and oral hygiene should be considered. In fact, the application of biomimetic hydroxyapatite [13], casein phosphopeptide-amorphous calcium phosphate [14], and calcium sodium phosphosilicate [15] showed promising results, and it should be evaluated in future reports involving cases with AI. Before starting the orthodontic treatment, the patient was provided with temporary crowns. The objective of the pre-orthodontic prosthesis was to facilitate the placement of orthodontic brackets. The structure of the enamel in AI is more porous, containing fewer minerals and more proteins. This does not allow a direct, effective bonding of the brackets to the tooth with a risk of enamel fracture. The orthodontic treatment prepares the arches for orthognathic surgery. In this case, the open bite correction was performed using the LeFort I impaction surgical technique and genioplasty. The surgical results were simulated on casts mounted on the articulator. However, 3-dimensional virtual planning of the surgery has also been previously reported as being easier to perform and giving more precise results [16]. Treating AI cases associated with severe open bite requires a long time to achieve the desired results [17].

In the present case, the treatment lasted four years and the final result was satisfactory for the patient, both functionally and esthetically. Although the length of such treatment is quite long, which is a disadvantage that could compromise the patient’s motivation, it is the ideal therapeutic alternative for achieving the result approved by the patient and the medical team.

## CONCLUSION

Our treatment strategy attempts to serve the patient needs in order to achieve better function and esthetics. Treatment not only restored function and esthetics, but also showed a positive psychological impact and thereby improved perceived quality of life.

## Data Availability

Data supporting this research article are available from the corresponding author or the first author upon reasonable request.
